# Matrix Metalloproteinases in Diabetic Kidney Disease

**DOI:** 10.3390/jcm9020472

**Published:** 2020-02-08

**Authors:** Nuria Garcia-Fernandez, Conxita Jacobs-Cachá, José María Mora-Gutiérrez, Ander Vergara, Josune Orbe, María José Soler

**Affiliations:** 1 Nephrology Department, Clinica Universidad de Navarra, 31008 Pamplona, Spain; jmora@unav.es; 2 Instituto de Investigación Sanitaria de Navarra (IdiSNA), 31008 Pamplona, Spain; josuneor@unav.es; 3 Nephrology Department, Hospital Universitari Vall d’Hebron, Universitat Autònoma de Barcelona, 08007 Barcelona, Spain; conxita.jacobs@vhir.org (C.J.-C.); vergara.ander@gmail.com (A.V.); 4 Nephrology Research Group, Vall d’Hebron Research Institute (VHIR), 08035 Barcelona, Spain; 5 Laboratory of Atherothrombosis, Program of Cardiovascular Diseases, Cima-Universidad de Navarra, CIBERCV, 31008 Pamplona, Spain

**Keywords:** matrix metalloproteinases, diabetic kidney disease, experimental models of diabetes, tissue inhibitors of metalloproteinases

## Abstract

Around the world diabetic kidney disease (DKD) is the main cause of chronic kidney disease (CKD), which is characterized by mesangial expansion, glomerulosclerosis, tubular atrophy, and interstitial fibrosis. The hallmark of the pathogenesis of DKD is an increased extracellular matrix (ECM) accumulation causing thickening of the glomerular and tubular basement membranes, mesangial expansion, sclerosis, and tubulointerstitial fibrosis. The matrix metalloproteases (MMPs) family are composed of zinc-dependent enzymes involved in the degradation and hydrolysis of ECM components. Several MMPs are expressed in the kidney; nephron compartments, vasculature and connective tissue. Given their important role in DKD, several studies have been performed in patients with DKD proposing that the measurement of their activity in serum or in urine may become in the future markers of early DKD. Studies from diabetic nephropathy experimental models suggest that a balance between MMPs levels and their inhibitors is needed to maintain renal homeostasis. This review focuses in the importance of the MMPs within the kidney and their modifications at the circulation, kidney and urine in patients with DKD. We also cover the most important studies performed in experimental models of diabetes in terms of MMPs levels, renal expression and its down-regulation effect.

## 1. Introduction

Diabetic kidney disease (DKD) is the main cause of chronic kidney disease (CKD) which is characterized by mesangial expansion, glomerulosclerosis, tubular atrophy, and interstitial fibrosis. Serveral mechanisms such as renin-angiotensin system disbalance, inflammation, proteinuria, and hypertension have been related to DKD progression from the early to the advanced stages of the disease [1].

The trademark of the pathogenesis of DKD is an increased extracellular matrix (ECM) accumulation causing thickening of the glomerular and tubular basement membranes, followed by mesangial expansion, sclerosis, and tubulointerstitial fibrosis. The ECM levels are regulated by a homeostatic balance between deposition and degradation of ECM components [2]. The matrix metalloproteases (MMPs) family are composed of zinc-dependent enzymes that are involved in the degradation and hydrolysis of ECM components [3]. The MMPs were discovered in the early 60s and are involved in the collagen degradation processes during the tadpole tail reabsorption [4]. Since then this family of enzymes and their roles have been expanded. Several MMPs are expressed in the kidney; in the nephron compartments, the vasculature and the connective tissue [5,6]. MMPs can contribute to diverse physiologic and pathological pathways in the kidney. Whereas the gelatinases, MMP-2 and MMP-9, have the ability to degrade type IV collagen, the major component of the glomerular basement membrane; the interstitial collagenases, MMP-1, MMP-8 and MMP-13, hydrolyze collagen I and III. Considering their important role in DKD, several studies have been performed in patients with DKD proposing that the measurement of their activity in serum or urine may become in the future markers of early DKD. Studies from diabetic nephropathy (DN) experimental models suggest that a balance between MMPs levels and their inhibitors is needed to maintain renal function. 

In this review, we describe the importance of the MMPs family within the kidney and their modifications at the circulation, kidney and urine in patients with DKD. We also cover the most important studies performed in experimental models of diabetes in terms of MMPs levels, renal expression and its inhibition effects. 

## 2. Matrix Metalloproteinases Family

MMPs are zinc-dependent proteases that degrade components of the extracellular matrix (ECM). MMPs were initially described as ECM regulatory proteins because of their ability to degrade ECM proteins like collagen, gelatin, laminin, aggrecan, fibronectin, elastin, and proteoglycans [7]. It was later observed that they can also process growth factors, cell-surface receptors, cytokines, and chemokines, as well as other MMPs, and proteases [8]. The activation or inactivation of biomolecules by MMPs cleavage points towards possible unexpected functions of these proteases beyond ECM remodeling [9]. Therefore, MMPs have a relevant physiological function and an altered expression or dysregulation leads to the development of several diseases such as chronic inflammatory diseases, vascular and renal diseases, diabetes, neurological disorders, and cancer [2,10,11].

All MMPs are multidomain enzymes that share a highly conserved core structure. They contain a signal peptide, which directs them to the secretory pathway; a prodomain with a cysteine residue to stabilize and inhibit a conserved HEXGHXXGXXH motif in the catalytic domain; a hinge or linker region; and a hemopexin-like domain that mediate protein-protein interactions and contributes to substrate specificity [7,12]. MMPs are classified into six groups based upon substrate and sequence homology: collagenases, gelatinases, stromelysins, matrilysins, membrane-type MMPs, and “other MMPs” [13,14,15]. Collagenases (MMP-1, -8, -13, and -18) cleave native collagens I, II, and III at a specific site. Gelatinases (MMP-2 and -9) cleave the denatured collagens (gelatins) and laminin, as well as some chemokines. Stromelysins (MMP-3, -10, -11, and -19) degrade a variety of substrates, including fibronectin, laminin, gelatin, and casein, and can cleave the prodomain of other MMPs activating them [13]. Matrilysins (MMP-7 and -26) are characterized by the lack of a hemopexin domain [15]. MMP-7 degrades not only ECM components such as laminin but also a large number of cell surface molecules, such as E-cadherin, Fas ligand and pro-α-defensin. Membrane-type MMPs (MT-MMPs; MMP-14, -15, -16, -17, -24, and -25) are structurally similar to the other classes of MMPs but they are anchored to the outside of the cell membrane by a transmembrane domain, or by a glycosylphosphatidylinositol residue [15] (Table 1). The remaining MMPs, MMP- 12, -20, -21, -23, -27, and -28, which do not fit into any of the above categories, are currently referred as “other MMPs”.

The role of MMPs in multiple physiological processes is controlled by different factors. In fact, dysregulation of MMPs has been associated to the development of pathological conditions. Regulation of MMP activity depends on transcriptional, epigenetic, and post-transcriptional mechanisms, proteolytic activation, post-translational modifications and extracellular inhibition [9]. MMPs expression is regulated at the transcriptional level, keeping these enzymes at low levels in normal physiological state. However, MMPs expression is induced in response to different stimuli when ECM remodeling is required. In addition, DNA methylation or histone acetylation are epigenetic mechanisms of MMP genes transcription. At the post-transcriptional level, MMP expression can be regulated by modulation of messenger RNA (mRNA) stability and micro RNA (miRNA)-based or maintaining the pro-enzymes in an inactive state [12]. MMPs normally remain inactive due to a “cysteine switch” motif located in the propeptide region that blocks the Zn^2+^ dependent catalytic region. Diverse proteolytic enzymes (serine proteases, furin, plasmin or even other MMPs) can activate the MMPs [14]. Oxidative stress or nitric oxide (NO) can also activate MMPs by destabilization of the “cysteine switch” resulting in active proMMPs [16]. Finally, the MMPs activity is also controlled by specific endogenous inhibitors such as tissue inhibitors of metalloproteinases (TIMPs). TIMPs share a similar structure to MMPs that fits into the active site of the MMP catalytic domain in 1:1 stoichiometric ratio [11]. Overall, the proteolytic activity of TIMPs depends on the relative concentration of the active enzymes and their inhibitors [17].

## 3. Matrix Metalloproteinases Pathways in the Kidney

Several MMPs are expressed in the kidney; in the nephron compartments, the vasculature and the connective tissue [5,6]. MMPs can contribute to diverse physiologic and pathological pathways in the kidney. Chronic kidney disease (CKD) is characterized by a progressive decline in renal function that end up with fibrosis at a histological level [18,19]. The pathological mechanism of fibrosis is complex as a series of molecular events lead to an excess of ECM deposition. In response to noxious stimuli, renal cells (glomerular, tubular, vascular or pre-existing infiltrated cells) secrete profibrotic and proinflammatory molecules that will promote the recruitment of inflammatory cells in a positive feedback mechanism. This inflammatory state leads to epithelial-to-mesenchymal transition (EMT) of the tubular cells, recruitment of fibrocytes and proliferation and dedifferentiation of fibroblasts to myofibroblasts. All these cell types produce and deposit ECM thus contributing actively to fibrosis progression [20,21,22]. The primary function of the MMPs is to degrade ECM components thus it seems obvious that MMP activation or decrease of TIMPs in the kidney would be beneficial but this relation is not that straightforward. Some studies sustain that down-regulation of MMPs activity or upregulation of TIMPs in the kidney could contribute to fibrosis [23,24]. MMP knockout mice models have shown increased fibrosis and accelerated kidney disease progression [25]. In contrast, several reports have shown that upregulation of MMPs promote fibrosis [5,26,27,28] maybe due to interaction with overexpressed TIMPs [27,29] or due to the capacity of MMP degradation products to induce EMT [5,30]. 

**Figure 1:**
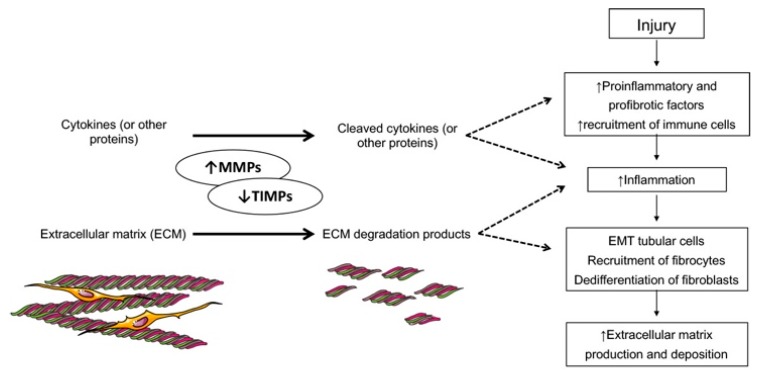
Matrix metalloproteinases/Tissue inhibitors of metalloproteinases (MMPs/TIMP) activity and function in the extracellular matrix turnover. In diabetes the majority of MMPs are upregulated contributing actively to all stages of renal fibrosis formation and progression: inflammation, fibrotic type cell recruitment, stimulation of tubular epithelial-to-mesenchymal transition (EMT) and extracellular matrix (ECM) production and deposition.

In a study performed by Tan et al., they exposed murine tubular epithelial cells to an activated macrophage conditioned medium. They demonstrated that exposure to this medium induced EMT in tubular epithelial cells, and that the use of MMP broad spectrum inhibitor reversed these changes [31]. They also identified that macrophage conditioned medium was rich in MMP-2 and MMP-9. Moreover, MMPs actively contribute to inflammation promoting proinflammatory pathways activation mediated by cytokines cleavage [32] and also MMP degradation products induce infiltration of immune cells [5]. In summary, under physiological conditions a regulated MMP activity is crucial for ECM turnover and tissue homeostasis but in a pathological context most MMPs are upregulated contributing actively to all stages of renal fibrosis progression: inflammation, fibrotic type cells recruitment, stimulation of tubular EMT and ECM production and deposition (Figure 1).

## 4. MMPs in Human Diabetic Kidney Disease

The activity of several MMPs is altered in human diabetic kidney disease (DKD). As explained before MMPs are not only involved in ECM remodeling, but also in the release of different growth factors such as tumor necrosis factor α (TNF-α), transforming growth factor β1 (TGF-β1), and others [33]. Therefore, this protein group dysregulation interferes with normal ECM turnover and may stimulate EMT and fibrosis by increasing the availability of previously mentioned growth factors [34]. In addition, the expression of these endopeptidases has not been well established in human kidney and there are species-dependent differences that hinder the characterization of these proteins (Table 1).

MMP-2 and MMP-9 are two of the proteinases that have been widely studied in human DKD. The concentrations and activity of both proteins are increased in urine of type 1 and 2 diabetic patients [35,36,37]. The increase in these MMPs is especially frequent in patients with albuminuria, and has been correlated with an established renal injury. However, other studies also demonstrated an increase in MMPs in normoalbuminuric patients that could indicate early renal involvement before the development of proteinuria [36,38]. The significance of the increase in plasmatic concentrations of these proteinases is controversial and may be more related to diabetic vascular damage and endothelial dysfunction than to kidney disease. Recently, two different isoforms of MMP-2 have been identified: the full length MMP-2 (FL-MMP-2) and the N-terminal truncated MMP-2 (NTT-MMP-2). The latter is an intracellular isoform and appears to be induced by an alternate promoter located in the first intron that is activated by oxidative stress. It has been related to the activation of the innate immune response and the subsequent inflammation. Expression of these two MMP-2 isoforms was increased in 25 kidney biopsies of type 2 diabetic patients as compared to healthy controls. Moreover, both proteinases co-localized in affected tubules and have been associated to mononuclear cell infiltration [39]. These findings support previous data where MMP-2 seemed to facilitate EMT and fibrosis by interfering with intercellular connections and releasing TGF-β1 [34]. Similarly, another group of MMPs, the stromelysins, have been related to tubular atrophy, interstitial infiltration and fibrosis in DKD. MMP-3 was found inversely correlated with mesangial expansion and glomerular damage, whereas it was increased in tubular atrophy and interstitial lesions [40]. Another proteinase, the MMP-7, is not expressed nor detected in healthy renal tubular epithelium. However, its upregulation has been described in different renal pathological states such as autosomal-dominant polycystic kidney disease and hydronephrosis [41,42]. Interestingly, in a study performed in 121 patients with type 2 diabetes, increased urinary MMP-7 levels were associated with all-cause mortality. However, no clear connection was found between these levels and end-stage renal disease (ESRD) development [43]. Other metalloproteinases have also been related to tubular lesions and interstitial fibrosis by releasing profibrotic factors. Among them, enhanced tubular expression of a disintegrin and metalloproteinase protein 17 (ADAM17) has been demonstrated in DN. This proteinase is responsible for TNF-α and amphiregulin (an epidermal growth factor receptor ligand) activation from its proligand substrates [44]. Moreover, the increase in ADAMs serum activity has been independently associated with CKD progression in males and an increased risk of cardiovascular events in CKD patients [45].

Specific MMPs are additionally responsible for other metalloproteinases activation. For instance, pro-MMP-2 is converted into its active form by MMP-24, a membrane-type metalloproteinase also known as MT5-MMP. In human DKD, there is an increased expression of this protein in the tubular epithelium, which correlates with a consequent MMP-2 elevation. Furthermore, proximal, distal and collecting tubules that where positive to MT5-MMP usually showed tubular atrophy as a probable result of DN progression [46]. An additional endopeptidase responsible for pro-MMP-2 activation is the MMP-14, of which membrane and soluble forms had been identified. In patients with type 1 and 2 diabetes these soluble forms are also increased in the urine [37].

TIMPs have also proven to be altered in human DKD. In some studies, circulating concentrations of TIMP-1 and TIMP-2 are decreased in patients with type 2 diabetes and diabetic nephropathy when compared to non-diabetic CKD or patients with diabetes alone [47]. In contrast, increases of serum and urinary concentrations of TIMP-1 were reported in patients with diabetes, and this increase was associated with greater glomerular lesions [48]. The same study that demonstrated increased MMP-3 expression also showed a higher TIMP-1 expression in tubules with atrophy, but both increases inversely correlated with stablished glomerular mesangial expansion [40]. Altogether, interstitial and tubular lesions, which usually correlate with glomerular lesions in DKD, seem more likely to be the source of urinary TIMP-1, as the inhibitor expression decreases in damaged glomeruli. 

A greater understanding of kidney MMPs is still needed to better delineate their pathophysiological role in DKD. Although most of these endopeptidases appear to be increased in human DN and apparently related to tubular atrophy and interstitial fibrosis, it is not certain if the activity and concentrations measured in the urine reflect what is really happening in renal tissue or are the consequence of an increased systemic activity [49]. Moreover, it is not yet established if metalloproteinases upregulation precedes fibrosis or if it is a consequence of this lesion. Some studies suggest that MMPs increases antedates the development of albuminuria and, therefore, a more established renal lesion [38,50]. Probably multiple pathological pathways that meet in diabetic nephropathy such as hyperglycemia, advance glycation end products (AGEs), oxidative stress or renin angiotensin system (RAAS) activation are involved in MMPs imbalance. Nevertheless, further MMPs characterization is needed to suggest the use of these proteinases as a possible DKD biomarker or therapeutic target. 

## 5. MMPs in Experimental Diabetic Nephropathy

Experimental studies have shown valuable insights into the potential role of MMPs in DN, demonstrating altered expression and enzyme activity in DN rodent models. Evidence supports that MMPs may have both a pathogenic and renoprotective effects. Moreover, the same MMP can play both roles, which underlines the complexity of MMP-system pathobiology [51]. Table 2 summarizes some of the most important experimental studies related to MMP-system performed in experimental models (in vivo and in vitro) of DKD.

Gelatinases (MMP-2 and -9) are the most widely studied MMPs in DN. Early compensatory increases and late decreases in MMP-2 expression and enzymatic activity have been reported in streptozotocin (STZ) diabetic rats [52]. Consistently, Mmp2 null mice worsened renal lesions namely urinary albumin excretion, accumulation of ECM in the glomeruli, atrophy and fibrosis in the tubulointerstitium of STZ-diabetic mice [25]. Han et al. observed an increase in MMP2 expression after 2 days of hyperglycaemia, although after 4 weeks MMP2 expression decreased as a response to the expression of TIMP-2 in glomeruli from Sprague-Dawley (S-D) rats [53]. These results suggest that an imbalance within the MMP-2 and TIMP-2 system, plays an important role in the pathogenesis of diabetic nephropathy. Singh et al. observed, after 5 days of hyperglycaemia, that mesangial cells presented a 25% reduction in MMP-2 activity, resulting in mesangial matrix accumulation mediated by angiotensin II type 1 (AT1) receptors via TGF-β1 [54]. Decreased glomerular MMP-2 expression accompanied augmented collagen-IV expression and glomerulosclerosis in advanced DN [55]. In contrast, increased expression and activity was found in glomeruli of diabetic rats in association with mesangial expansion [56]. Proximal tubular cells also showed enhanced MMP-2 activity induced by advanced glycation end products (AGE) and angiotensin-II [57]. McLennan et al. analysed renal gene expression and activity of MMP-2 and MMP-9 at 24 weeks, observing increased Mmp2 expression but decreased MMP-2 enzymatic activity and Mmp9 down-regulation. These changes were attenuated by renin-angiotensin system (RAS) inhibition [58]. Recently, two different isoforms of MMP-2 were identified in diabetic models showing differentially distribution in renal tissue [59]. An interesting finding on genetic susceptibility to DKD was described by Fornoni et al., who analysed mesangial cells from murine strains resistant (B6) and susceptible (ROP) to sclerosis, mimicking patients who develop DKD despite an adequate glycaemic control. ROP-mice presented basal elevated levels of MMP-2, which increased even more in the presence of hyperglycaemia, although remained elevated when glycaemia was reduced. B6-mice presented an increment on MMP-2 when exposed to hyperglycaemia but returned to baseline levels when glycaemia was normalized. These findings suggest a potential role of MMP-2 in the genetic susceptibility to develop DN [60,79]. Analysing a type 2 diabetes rodent model, it has been shown that renal Mmp9 expression was augmented compared with non-diabetic mice [61]. Bai et al. reported that short- or long-term exposure to hyperglycaemia may produce diverse effects on MMP-9. After 2-3 days of hyperglycaemia, an enhanced expression and activity was related with decreased collagen-IV synthesis, while hyperglycaemic-exposure for more than 5 days revealed a down-regulation on MMP-9 expression and activity in podocytes [62]. A recent work, found no differences on MMP-2 and MMP-9 expression in high-fat high sucrose diet-induced diabetes, which presented only minimal interstitial alteration [63]. Urinary MMP-2 and MMP-9 enzymatic activities were increased in type 1 diabetic rodents, and urinary neutrophil gelatinase-associated lipocalin (NGAL)/MMP-9 ratio and MMP-9 activities augmented before the onset of albuminuria [50]. Furthermore, urinary MMP-9 excretion showed positive correlation with albuminuria in diabetic rats, and down-regulation was observed after simvastatin treatment, together with attenuation of glomerular disease [64]. Increased MMP9 staining was observed in glomerular parietal epithelial cells from Zucker diabetic Fatty rats, which was also associated with podocyturia [65]. In fact, increased MMP-9 overexpression and activity induced podocyte dedifferentiation and disruption of junction integrity [66]. MMP-9 has also been inversely correlated with miR-21 expression which plays a role on renal fibrosis on DKD [67].

Referring to matrilysins, McLennan et al. analysed mesangial cells of STZ-induced diabetic rats, observing a down-regulation on MMP-7 expression and increased fibronectin accumulation [68]. Membrane-type MMPs may also play a role in DN. Besides to modulate other MMPs, MT1-MMP (MMP-14) has shown to be up-regulated preceding the manifestation of glomerular pathologic lesions in type 2 diabetic rats [69]; although decreased expression has been reported in glomeruli of long-term diabetic mice [70]. Meprin also seems to have a protective effect on DKD [71]. Relative to stromelysins, our group recently demonstrated glomerular overexpression of MMP10, in podocytes and juxtaglomerular apparatus, in early stages of DN. Interestingly, MMP10 overexpression was prevented with telmisartan administration. These changes were observed despite the absence of significant histological lesions, moreover a correlation was observed between glomerular MMP10 and albuminuria [72]. Consistently, Mmp-10 null diabetic mice presented fewer mesangial expansion, renal macrophage infiltration and renal function impairment [73]. These findings may support a deleterious glomerular effect of MMP-10 on DN, which may be related with the RAS pathway. Another study observed decreased glomerular expression of MMP-3 and MMP-1 in diabetic rats [74]. Unlikely to enalapril [74], insulin treatment ameliorated these changes [75]. MMP-1 expression and serum levels were also down-regulated in alloxan induced-diabetic mice [76,77], while local delivery of Mmp-1 into mice kidneys prevented diabetic renal fibrosis [78]. 

Analysing the mechanisms by which MMPs may be altered in DN, data suggest that inflammatory pathways may regulate MMPs expression, however some MMPs may also modulate the expression of inflammatory mediators [80]. It has been reported that insulin-like growth factor 1 (ILGF-1) decreases MMP expression [81,82,83]. TGF-β has shown to upregulate MMP-2 [84]. Endothelin also produces an inhibitory effect on MMP-2 and MT1-MMP [85]. Podocyte Vegf₁₆₄ overexpression down-regulated MMP-2 expression resulting in advanced DN [86]. Hydrogen sulphide enzymes can attenuate MMP-9 expression in the diabetic kidney and mitigate adverse ECM accumulation [87]. IL-20 may play a pathologic role in early DN through upregulation of MMP-9 [88].

Experimental studies have shown that dysregulation of MMPs expression/activity may play a role in DKD through different pathways, underlying the high complexity of the MMP-system. Therefore, in order to understand diverse outcomes from different studies, it is important to take into account: the high homology among MMPs, their diverse locations, diverse substrate specificity, regulators, the discrepancies between in vitro and in vivo models, as well as the different roles they play according to the time of evolution of the diabetes (Table 3). Taken together, MMPs expression and activity may vary in DN, and their pathobiological system is still very confounding. However, an important role seems to be played by both MMPs and their inhibitors in the hallmark of DKD.

## 6. Tissue Inhibitors of Metalloproteinases and Modulators in the Kidney

TIMPs are specific endogenous inhibitors of metalloproteinases and frequently, their transcriptional regulation is related to MMPs. In fact, MMPs may modulate TIMP signaling by sequestration. Four TIMPs have been identified (TIMP-1, TIMP-2, TIMP-3 and TIMP-4). Although MMPs inhibition is the main role of TIMPs, they can also participate in metalloproteinase activation and, together with MMPs, in other biological processes such as cytokine production, inflammation, migration, cell proliferation and apoptosis [7]. All of these processes have been shown to have potential pathogenic pathways in tissue damage [99]. The role of TIMP in ECM turnover regulation can be different depending on the specific metalloproteinase inhibited and local tissue factors [17]. These multiple functions and complex interactions between TIMPs and MMPs explain how difficult is to define their pathogenic role in different pathologies and diseases [5]. 

Although renal expression TIMPs has not been completely characterized, all TIMPs, apart from TIMP-4, are expressed in healthy kidney. Human glomeruli express TIMP-1 and TIMP-2, and the upregulation of both has been demonstrated in glomerulosclerosis [100]. Distal convoluted tubular (DCT) expression of TIMP-2 and TIMP-3 has been described in normal kidney [5]. Disregulation of MMPs/TIMPs is implicated in excessive accumulation of ECM in CKD. In fact, suppression of MMP activity and enhanced TIMP expression are associated to fibrosis progression in CKD. Although TIMP-1 overexpression occurs in fibrosis and can promote it independently of MMP inhibition, TIMP-1 deficiency cannot prevent fibrosis due probably to other TIMP compensatory upregulation [101]. TIMPs deletion has suggested a possible protective role of TIMP-3 and fibrotic role of TIMP-2 in renal fibrosis mice models [102]. 

Dysregulation of MMP/TIMP has been described in clinical studies performed in patients with DKD. In patients with DKD, decreases in serum TIMP-1 and TIMP-2 levels, and increases in serum and urine TIMP-1 levels have been described in association with worsening glomerular lesions [47,72]. In contrast, in experimental DN models, decreases in some MMPs such as MMP-2 [58] and increase of TIMP-1 [58] and TIMP-2 expression [53] have been associated to DN progression. TIMP-3 has been found to be down-regulated in diabetic nephropathy and increased TIMP-3 expression shows a renoprotective role for DN progression [103]. Furthermore, its down-regulation is associated with increased renal fibrosis [6,104].

Potential interventions to attenuate DKD involve increasing MMP-2 activity and TIMP-3 expression and/or inhibiting MMP-9, TIMP-1 and TIMP-2 expression. It has been described the positive effect of the angiotensin-converting enzyme (ACE) inhibitors, estrogens and the peroxisome proliferator-activated receptor-γ agonist in TIMP and MMPs in different models of experimental DN. ACE inhibition attenuated MMP-9, decreased TIMP-1 mRNA, and increased TIMP-2 expression in STZ-diabetic rats [58,105]. Hepatocyte growth factor (HGF) gene therapy also inhibits TIMP-1 expression in STZ-diabetic rats [106]. Fluorofenidone inhibited TIMP-1 expression in the renal cortex from db/db mice (type 2 diabetes) [107]. Dencichine and hyperoside are traditional herbal medicines that ameliorated the renal dysfunction in DN experimental models. Specifically, they increased the MMP-9/TIMP-1 ratio that is exacerbated after the oral dencichine, and subsequently decreases the degradation of ECM [108]. Hyperoside decreased fibrosis by suppressing TIMP-1 expression and promoting MMP-9 expression [109]. Iron chelation by oral deferiprone promoted MMP-9 expression and decreased TIMP-1 in diabetic rats [110]. In addition, berberine decreased MMP-9 and TIMP1/2 levels and increased MMP2 expression in diabetic rats [111]. miR-21 depletion inhibited the progression of DN by promoting TIMP-3 overexpression and inhibiting TIMP-1 expression in STZ-diabetic rats [6,89]. 

## 7. Conclusions

Taken together MMPs and TIMPs are involved in the development and progression of DN. In patients with diabetes different MMPs such as MMP-2 and MMP-9 are increased in urine, especially in patients with albuminuria and established renal injury. Moreover, it has been speculated that their upregulation may precede early diabetic nephropathy and albuminuria. Research in animal models suggested that the equilibrium between MMPs and its inhibitors is altered in DN. However, experimental studies have also shown contradictory results. These results manifest the complex regulations that exist between different MMPs, TIMPs, and other proinflammatory and profibrotic factors. MMPs and TIMPs probably modify their expression and localization throughout the renal diabetic involvement, and other elements such as drugs or associated pathologies influence these changes, which may help to explain the variability of the observed results to date. Therefore, further studies are needed to improve the characterization and knowledge of MMPs imbalance in diabetic kidney disease.

## Figures and Tables

**Table 1:** Different matrix metalloproteinases (MMPs) implied in diabetic kidney disease (DKD) and their inhibitors. MMPs and tissue inhibitors of metalloproteinases (TIMPs) expression and characterization is still complex and vary between species.

Group	MMP	Other Names	Molecular Weight	Substrates	Renal Location
**Collagenases**	MMP-1	Interstitial collagenase	54 kDa. Cleaved in different isoforms that range from 22 to 27 kDa.	Type I, II, III, VII and X collagens.	Rats: glomeruli
	MMP-13	Collagenase 3	54 kDa.	Type I, II, III, IV, X and XIV collagens and fibronectin.	Rats: glomeruli.
**Gelatinases**	MMP-2	Gelatinase A	Range from 65 to 72 kDa. Different isoforms.	Type IV, V, VII and X collagens, type I gelatin and elastin.	Rats/Mice: glomeruli, and proximal and distal tubules. Monkey: proximal and distal tubules. Human: tubules (in pathological conditions).
	MMP-9	Gelatinase B	92 kDa.	Type IV and V collagens and type I and V gelatins.	Rats/Mice: glomeruli and tubular cells. Monkey: proximal and distal tubules.
**Stromelysins**	MMP-3	Stromelysin-1	54 kDa.	Type III, IV, IX and X collagens, type I, III, IV and V gelatins, proteoglycans, fibronectic and laminin.	Rats: glomeruli. Monkey: proximal and distal tubules. Human: glomeruli and tubules.
	MMP-10	Stromelysin-2	62 kDa	Type III, IV and V collagens, gelatin and elastin. Enhances tissue plasminogen activator.	Mice: glomeruli and juxtaglomerular apparatus.
**Matrilysins**	MMP-7	Matrilysin	30 kDa.	Type I, III, IV and V gelatins, proteoglycans, fibronectin, laminin, E-cadherin and entactin.	Rats: glomeruli. Human: distal tubules and collecting duct (in pathological conditions).
**Membrane-type MMPs**	MMP-14	MT1-MMP	Range from 40 to 80 kDa. There are membrane and soluble forms.	Progelatinase A (pro-MMP-2) and MMP-15.	Rats/mice: glomeruli.
	MMP-24	MT5-MMP	64 kDa.	N-cadherin (CDH2), progelatinase A (pro-MMP-2), proteoglycans and fibronectin.	Human: proximal and distal tubules, collecting duct and loop of Henle.
**Other MMPs**	ADAM17	TACE	93 kDa.	pro-TNF-α, pro-HER ligands and other proligand substrates.	Human: proximal and distal tubules (in pathological conditions).
	MEP1B	Meprin A subunit beta	80 kDa.	Type I and III procollagens, FGF-19, VEGF-A, IL-1β, IL-18, ADAM10, E-cadherin and others	Mice: proximal tubule and juxtamedullary region.
**Tissue inhibitors of MMPs**	TIMP-1	None	23 kDa.	Inhibits MMP-1, MMP-2, MMP-3, MMP-7, MMP-8, MMP-9, MMP-10, MMP-11, MMP-12, MMP-13 and MMP-16.	Rats/mice: glomeruli. Human: glomeruli and tubules.
	TIMP-2	None	24 KDa.	Inhibits MMP-1, MMP-2, MMP-3, MMP-7, MMP-8, MMP-9, MMP-10, MMP-13, MMP-14, MMP-15, MMP-16 and MMP-19	Not clearly defined.
	TIMP-3	None	24 KDa.	Inhibits MMP-1, MMP-2, MMP-3, MMP-7, MMP-9, MMP-13, MMP-14 and MMP-15.	Not clearly defined.

MMP: metalloproteinase, MT-MMP: membrane-type metalloproteinase, TACE: tumor necrosis factor-alpha converting enzyme, ADAM: a disintegrin and metalloproteinase, HER: human epidermal growth factor receptor, FGF-19: fibroblast growth factor 19, MEP1B: Meprin A subunit beta, VEGF-A: vascular endothelial growth factor A, IL-1β: interleukin 1 beta, IL-18: interleukin 18, TIMP: tissue inhibitor of metalloproteinases.

**Table 2:** Experimental murine studies involving different metalloproteinases.

MMP	*In Vivo/in Vitro*	Species (Model)/Cell Type	Condition Studied	Age/Exposure Time	Outcome	Ref
MMP-2	*In vivo* *In vitro*	BN rats (STZ)/Cortex & medulla Podocytes *	Gene expr. Enz. activity	6 wks	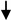	[52]
MMP-2	*In vivo*	C57BL/6J (STZ)/Renal cortex	Gene & Protein expression Enz. activity KO-*Mmp2*	16 wks	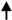 compensatory KO-*Mmp2* = renal injury	[25]
MMP-2	*In vivo/* *In vitro*	S-D rats (STZ)/Glomeruli & Tubules	Protein expr.	2 days 4 wks	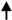 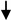	[53]
MMP-2	*In vitro*	S-D rats (HG) Mesangial cells	Enz. activity & Protein expr.	5 days	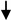	[54]
MMP-2	*In vivo*	ICER Iγ mice/Glomeruli	Protein expr.	40 wks	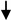	[55]
MMP-2 MT1-MMP	*In vivo/* *In vitro*	OLETF Rats/Glomeruli	Gene & Protein expr. Enz. activitiy	40–50 wks	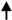	[56]
MMP-2	*In vivo/* *In vitro*	S-D rats (STZ)/Proximal tubular c.	Gene expr. Enz. activity	32 wks	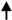 Induced by AGE & Ang-II Attenuated by Ramipril	[57]
MMP-2 MMP-9	*In vivo*	S-D rats (STZ)	Gene expr. Enz. activity	24 wks	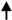 *Mmp-2*, 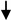 *Mmp-9* 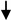 MMP-2 activity Attenuated by perindropil	[58]
MMP-2	*In vivo*	C57/BL6 (STZ) db/db mice/Cortex & medulla	Gene & Protein expr.	4–24 wks	NTT-MMP-2 FL-MMP-2	[59]
MMP-2	*In vitro*	B6 & ROP mice/Mesangial cells (HG)	Gene expr. Enz. activity	5–10 wks	**B6 mice:** 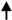 Hyperglycemia 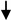 Normoglycemia **ROP mice:** 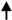 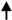 Hyperglycemia 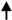 Normoglycemia	[60]
MMP-9	*In vivo*	Kkay mice/Glomeruli	Gene & Protein expr.	16 wks	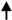	[61]
MMP-2 MMP-9	*In vitro*	Podocytes * (HG)	Gene expr.Enz. activity	2–3 days 5–10 days	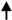 MMP-9, **Ø** MMP-2 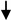 MMP-9, **Ø** MMP-2	[62]
MMP-2 MMP-9	*In vivo*	C57BL/6 mice (High-fat high sucrose diet)	Protein expr.	21 wks	**Ø**, Minimal Interstitial lesions None glomerular lesions	[63]
MMP-2 MMP-9	*In vivo*	C57BL6J & S-D (STZ)/Urine & Glomeruli	Urinary Enz. Activity Protein Expr.	16 wks	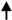	[59]
MMP-9	*In vivo*	Wistar rats (STZ)	Urinary levels Gene expr.	8 wks	Positive correlation with albuminuria	[64]
MMP-9	*In vivo* *In vitro*	Zucker diabetic fatty rats/Glomeruli S-D rats/Podocytes (HG)	Protein expr. Enz. activity Urinary activity Protein expr. Enz. activity	20–28 wks 24–48 h	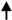 Correlated with podocituria and albuminuria 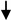	[65]
MMP-9	*In vivo* *In vitro*	FVB mice (STZ)/glomeruliPodocytes* (#)	Protein expr. Enz. activity KO-*Mmp9* MMP-9 overexpression	24 wks 14 days	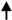 Co-stained wih nephrin KO-*Mmp9* = Fewer renal lesion Podocyte dedifferentiation Interrupt cells junction integrity Stimulate GBM synthesis	[66]
MMP-9	*In vivo*	Kkay/glomeruli & tubular cells	Gene & Protein expr.	8–20 wks	Negatively correlatedwith miR-21	[67]
MMP-7	*In vivo*	S-D rats (STZ)/Glomeruli	Gene expr. Enz. activity	32 wks	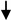 exacerbated by aminoguanidine^+^	[68]
MMP-2 MMP-9 MT1-MMP	*In vivo*	Goto-Kakizaki rats /cortex & medulla	Protein expr. Enz. activity	18 wks	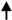 preceding glomerular lesions	[69]
MT1-MMP	*In vivo*	S-D rats (STZ)/Glomeruli	Protein expr. Inmunogold	4–48 wks	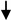	[70]
Meprin	*In vivo*	C57BL/6 (STZ)	Gene & Proteinexpr. KO-*Meprin*	12 wks 18 wks	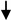 KO-*Meprin* = renal injury	[71]
MMP-10	*In vivo*	(db/db)/podocytes & juxtaglomerular	Gene expr.	8–16 wks	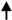 , attenuated by telmisartan	[72]
MMP-10	*In vivo*	C57BL/6 (STZ)	KO-*Mmp10*	17 wks	KO-*Mmp10* = Fewer renal lesion	[73]
MMP-3 MMP-1	*In vivo*	S-D rats (STZ)/glomeruli	Gene expr.	4–24 wks	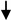 , Prevented by Insulin	[74,75]
MMP-1	*In vivo*	Kunming mice (alloxan) (BGF)	Gene & Protein expression	8 wks	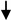	[76]
MMP-1	*In vivo*	Albino rats (alloxan)/circulating levels	Serum levels	14–42 days	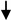	[77]
MMP-1	*In vivo*	C57BL/6 (STZ)/renal cortex	*Mmp1 DNA*	28 days	Prevented diabetic renal fibrosis	[78]

Abbreviations: 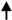 increase/upregulation, 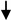 decrease/down-regulation, **Ø** no changes, BN: Brown Norway, STZ: streptozotocin-induced diabetes, HG: hyperglycaemia exposure, B6: B6SJLF1/J sclerosis-resistant mice, ROP: ROP/Le- Es1b/ES1a sclerosis-prone mice, Alloxan: alloxan-induced diabetes, S-D: Sprague-Dawley rats, ICER Iγ: ICER Iγ transgenic mice, OLETF: Otsuka Long-Evans Tokushima Fatty rats, C57BL/6: C57 black 6 strain, KO-Mmp2: MMP-2 knockout mice, FVB: friend leukemia virus B strain, Goto-Kakizaki rats: non-hypertensive model of type 2 diabetes, (*) immortalized mouse podocyte cell line, Ang-II: Angiotensin II, gene expr.: gene expression determination, enz. activity: enzymatic activity determination, wks: weeks, ^+^aminoguanidine: AGE formation inhibitor, BGF: blood glucose fluctuation model, #: podocytes were treated with D-glucose, advanced glycoprotein end-product bovine serum albumin, angiotensin II, VEGF, TNF-alfa and TGF-beta1.

**Table 3:** Crucial considerations to understand contradictory results when mimicking the experimental outcomes to human studies.

**I.**	Insulin [89], RAS inhibition [74,81,90], oral antidiabetics [91,92] and diverse pharmacological drugs [63,93,94,95] may have an effect on MMPs expression/activity. Murine models based on STZ or Alloxan-induced diabetes imitate a hypoinsulinemic state (T1D), while those which T2D (db/db, Kkay, OLETF) are characterized by hyperinsulinism. Moreover, the RAS inhibition is commonly used in diabetic patients with early DKD. Renin-angiotensin activation could be influenced according to the murine model employed in the study (hypertension, obesity, salt-intake).
**II.**	Macrophages infiltration could precede the appearance of DN signs [96]. But also, during diabetes exists a greater predisposition to urinary tract infections, which may also stimulate macrophage migration and could be unnoticed if screening analysis or meticulous histological inspection is not performed. The presence of macrophages due to renal infection may modify the MMPs outcomes of the study.
**III.**	MMPs expression/activity may vary depending on the type of the renal cell evaluated (i.e., proximal/distal tubule, mesangial, podocyte cells) [6]. Also, different conditions and time of evolution of disease [57] are presented according to *in vitro* or *in vivo* models. Moreover, it has been reported that murine strains may also show certain discrepancies in MMPs outcomes according to the genetic background [59].
**IV.**	Some MMPs share a great homogeneity, therefore certain molecular techniques may interfere in the results due to different sensitivities of the assay systems employed [97,98]. For example, MMP-3 shares 82% homology with MMP-10 at the protein level, which may result in the recognition of both proteins.
**V.**	Clinical studies, as consequence of limitations for *in vivo* renal histological analysis, usually analyse the urinary activity/levels rather than renal expression. However these conclusions could present a wide variety of pathogenic meanings (i.e., enhanced intrarenal production vs increased tubular shedding of the protein).
